# Pan-chloroplast genomes for accession-specific marker development in *Hibiscus syriacus*

**DOI:** 10.1038/s41597-024-03077-7

**Published:** 2024-02-27

**Authors:** Sangjin Go, Hyunjin Koo, Minah Jung, Seongmin Hong, Gibum Yi, Yong-Min Kim

**Affiliations:** 1 Plant Systems Engineering Research Center, Korea Research Institute of Bioscience and Biotechnology (KRIBB), Daejeon, 34141 Republic of Korea; 2 Department of Bioinformatics, KRIBB School of Bioscience, Korea University of Science and Technology (UST), Daejeon, 34141 Republic of Korea; 3 Department of Bio-Environmental Chemistry, College of Agriculture and Life Sciences, Chungnam National University, Daejeon, 34134 Republic of Korea; 4 Digital Biotech Innovation Center, Korea Research Institute of Bioscience and Biotechnology (KRIBB), Daejeon, 34141 Republic of Korea

**Keywords:** Genetics, Plant sciences

## Abstract

*Hibiscus syriacus* L. is a renowned ornamental plant. We constructed 95 chloroplast genomes of *H. syriacus* L. cultivars using a short-read sequencing platform (Illumina) and a long-read sequencing platform (Oxford Nanopore Technology). The following genome assembly, we delineate quadripartite structures encompassing large single-copy, small single-copy, and inverted repeat (IRa and IRb) regions, from 160,231 bp to 161,041 bp. Our comprehensive analyses confirmed the presence of 79 protein-coding genes, 30 tRNA genes, and 4 rRNA genes in the pan-chloroplast genome, consistent with prior research on the *H. syriacus* chloroplast genome. Subsequent pangenome analysis unveiled widespread genome sequence conservation alongside unique cultivar-specific variant patterns consisting of 193 single-nucleotide polymorphisms and 61 insertions or deletions. The region containing intra-species variant patterns, as identified in this study, has the potential to develop accession-specific molecular markers, enhancing precision in cultivar classification. These findings are anticipated to drive advancements in breeding strategies, augment biodiversity, and unlock the agricultural potential inherent in *H. syriacus*.

## Background & Summary

*H. syriacus*, commonly known as rose of Sharon, is a fast-growing deciduous shrub belonging to the Malvaceae family and is renowned for its diverse applications, including culinary, ornamental, and medicinal uses^1^. Its wide range of flower colors makes it an attractive choice for decorative landscaping^2,3^. In North American countries, it has gained immense popularity as a garden tree due to its versatile properties^4^. However, breeding *H. syriacus* presents significant challenges due to its self-incompatibility, resulting in most landraces being natural hybrids^5^. Consequently, there have been limited reports of breeding trials aimed at developing polyploidy plants^4,6^. In Korea, breeding advancements have been achieved through methods such as the propagation of naturally occurring mutants, inter-generic crossings, and the induction of mutations using gamma-ray irradiation^6–9^. The complexities of breeding *H. syriacus* highlights the importance of elucidating the phylogenetic relationships among its cultivars to establish a breeding system capable of generating F1 hybrids.

Given the challenges in *H. syriacus* breeding, the utilization of chloroplast genomes represents a strategic approach due to their unique features. These organelles are typically maternally inherited, except in some gymnosperms where inheritance is paternally directed. Chloroplast genomes contain non-recombinant sequences and are usually inherited in a uniparental manner, allowing for lineage tracing through the maternal line and minimizing uncertainties associated with biparentally inherited nuclear genomes^10–13^. Furthermore, the high conservation of the chloroplast genome, including gene repertories and structures, enables comparative analyses that offer clear insights into the evolutionary trajectories and phylogenetic relationships among cultivars^14–16^. Previous studies on *Atractylodes* species and *Panax ginseng* demonstrated that even with low divergence, unique polymorphic chloroplast-derived markers could be developed to distinguish inter- and intra-species differences, respectively^11,17–22^. This highlights the potential applications of chloroplast genomes in the development of highly species-specific molecular markers, even at the intra-species level, thereby overcoming challenges posed by minimal genetic divergence. Nevertheless, the majority of studies on *Hibiscu*s chloroplast genomes have predominantly focused on the taxonomic level of genus, leaving in-depth intra-species studies relatively unexplored^10,23–25^. Given the breeding challenges of *H. syriacus* outlined earlier, comparative studies at the intra-species level are not only crucial but indispensable. Developing more molecular markers at the intra-species level is essential to gain unparalleled insights into the evolutionary trajectory and contribute to the precise taxonomic classification of *H. syriacus*^26–28^.

In this study, we generated 94 *H. syriacus* chloroplast genomes using a short-read sequencing platform (Illumina) and 1 genome using a long-read sequencing platform (Oxford Nanopore Technology). Subsequent pangenome analysis of these 95 *H. syriacus* chloroplast genomes revealed a high degree of conservation in the majority of genome sequences, while also identifies unique cultivar-specific variant patterns. A total of 193 single-nucleotide polymorphisms (SNPs) and 61 insertions or deletions (Indels) were identified, highlighting their potential applications as intra-species molecular markers^29^. The development of molecular markers utilizing these regions will play a pivotal role in achieving precise classification among *H. syriacus* cultivars and establishing refined breeding strategies. Moreover, these results will offer essential insights for species conservation, biodiversity enhancement, and the exploration of the agricultural and ornamental potentials of *H. syriacus*.

## Methods

### Plant materials and sequencing

*H. syriacus* cv. Gangneung was used for long-read-based chloroplast genome assembly^30^. A core collection of *H. syriacus* from the National Institute of Forest Science was utilized for short-read-based chloroplast genome assembly. Genomic DNA was extracted from fresh leaf tissues of *H. syriacus* plants using the standard cetyltrimethylammonium bromide method^31^.

The quantity and quality of genomic DNA were assessed using a Nanodrop spectrophotometer with a quality cut-off at an OD_260/280_ ratio of 1.8–2.0 and a Qubit dsDNA HS Assay Kit (Thermo Fisher Scientific, Massachusetts, USA). Following quality assessment, the DNA was used to generate libraries with an average insert size of 550 bp. Paired-end sequencing was performed to obtain 150-bp sequences at both ends using an Illumina NovaSeq. 6000 platform (Illumina Inc., San Diego, CA, USA).

### Genome assembly and annotation

For long-read assembly, the generated reads^30^ were aligned to a reference chloroplast sequence obtained from prior research^32^, using minimap2 (v2.22) with default parameters^33^. Reads with a mapping coverage exceeding 80 were extracted using Seqtk (https://github.com/lh3/seqtk) v1.3. These extracted reads were then assembled into a pseudo-molecule using Flye (v.2.9)^34^ and subsequently polished using NextPolish (v1.4)^35^ to correct base errors arising from noisy long reads.

For short-read assembly, Trimmomatic (v0.39)^36^ was used to trim adapters and eliminate low-quality sequences from the raw reads to enhance read quality. The trimmed reads were then aligned to the reference chloroplast genomes obtained from prior studies^37–44^, using the Burrows–Wheeler alignment (v0.7.17) tool^45^ (Table 1). The mapped reads were assembled using NOVOPlasty (v4.3.1)^46^, which employed a 39 k-mer and default RUBP sequences as seeds for chloroplast assembly^47,48^. The contigs generated by NOVOPlasty were ordered and merged into a single pseudo-molecule according to the reference chloroplast genome sequence.

**Table 1:** Reference chloroplast genomes for mapping.

Species	Accession ID
*Gossypium arboretum*	NC_016712.1
*Gossypium hirsutum*	NC_007944.1
*Gossypium raimondii*	NC_116668.1
*Hibiscus cannabinus*	NC_045873.1
*Hibiscus rosa-sinensis*	NC_042239.1
*Hibiscus syriacus*	NC_026909.1
*Hibiscus taiwanensis*	NC_054167.1
*Hibiscus trionum*	NC_060636.1

**Fig. 1:**
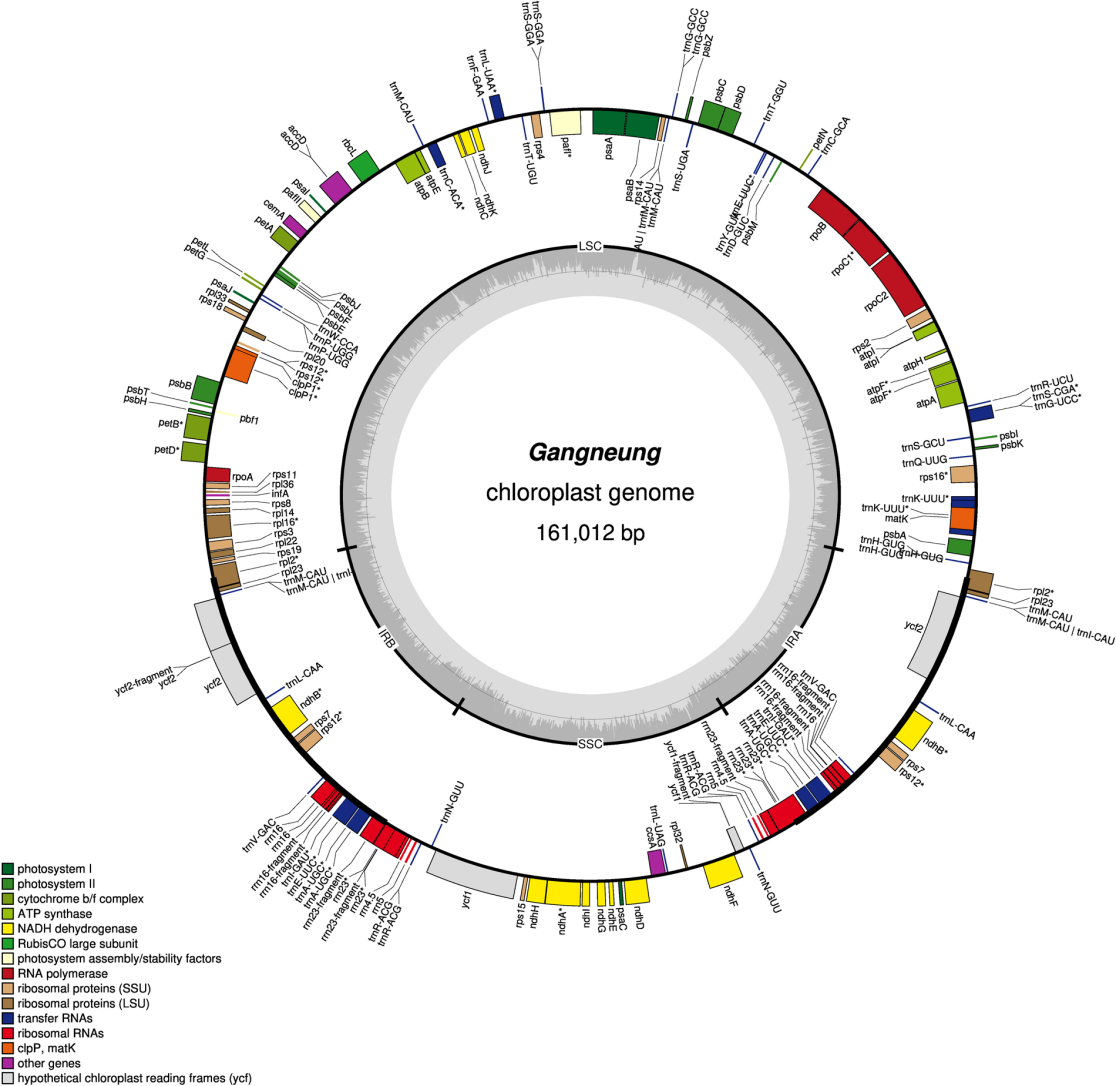
Circular map of the chloroplast genome in *H. syriacus* var. Gangneung. The center of the plot displays the cultivar name and genome length. The inner grey circle represents the GC content proportion in each region, with the line representing 50%. Genes located outside the outer circle are transcribed counterclockwise, and those inside the circle are transcribed clockwise. Genes with different functional annotations are differentiated by color.

Genome annotation was performed using the GeSeq platform, which provides rapid and accurate annotation of organellar genomes^49^. We employed BLAT^50^, Chloë (v0.1.0), and HMMER^51^ to annotate coding sequences and rRNA, and ARAGORN (v1.2.38)^52^ and tRNAscan-SE (v2.0.7)^53^ to annotate tRNA. Annotation accuracy was validated against *H. syriacus* var. Baekdansim^30^, and any discrepancies were manually curated. The circular map representation of the chloroplast genome was generated using OGDRAW (v1.3.1)^54^ (Fig. 1).

### Chloroplast genome alignment and pan-chloroplast genome-graph construction

To validate the genome assembly, we employed the chloroplast genome of *H. syriacus* var. Gangneung, constructed using long-read sequencing, as a reference for multiple sequence alignment. Sequence alignment was performed using MAFFT^55^ with default parameters. Subsequently, pairwise alignments of the chloroplast genomes were generated using MUMmer4^56^.

**Fig. 2:**
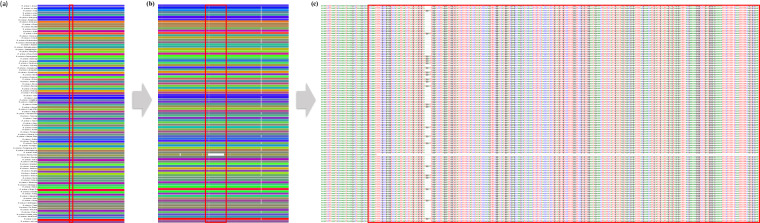
Pan-chloroplast genome-graph for 95 *H. syriacus* cultivars. (**a**) The pan-chloroplast genome-graph represents all 95 *H. syriacus* cultivars with the total chloroplast genome scale. (**b**) An enlarged view of the pan-chloroplast genome graph highlighting a region of the largest variation identified in *H. syriacus* var. Russian violet, indicated by red bars. (**c**) Multiple sequence alignment for the largest variation site among the 95 *H. syriacus* varieties.

To construct a pan-chloroplast genome-graph encompassing 95 *H. syriacus* genomes, we utilized the Minigraph-Cactus Pangenome Pipeline (v2.6.8)^57^. The integration process involved the iterative addition of the remaining 94 genomes with the reference chloroplast genome. Precise base-level alignments were achieved with the Cactus-pangenome tool using the parameters “--giraffe --fa --bz --viz.” From this comprehensive graph, we employed the Cactus-graphmap (v2.6.8) tool to map the graph utilizing the default parameters. We identified a total of 193 SNPs and 61 Indels across the entire genomes, observations that offer significant potential for the future development of intra-species molecular markers^29^. Overall, *H. syriacus* cultivars exhibit similarity across all genomic regions. However, for *H. syriacus* var. Russian Violet, a notable divergence in similarity was observed in the regions spanning 59,000 bp to 62,000 bp (Fig. 2).

### Comparative genomic analysis in 95 *H. syriacus* chloroplast genomes

**Fig. 3:**
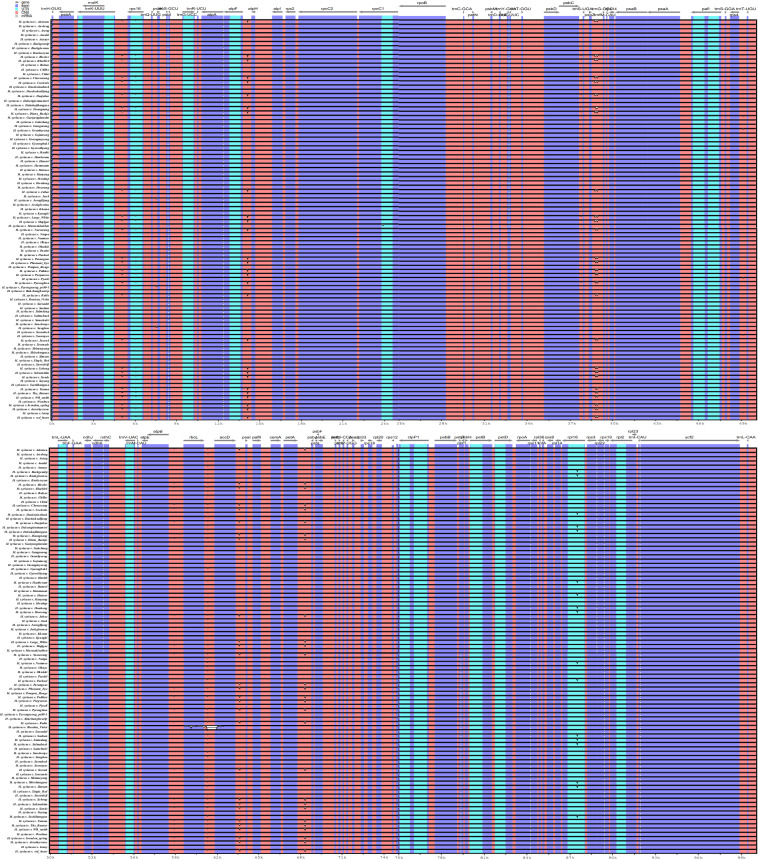

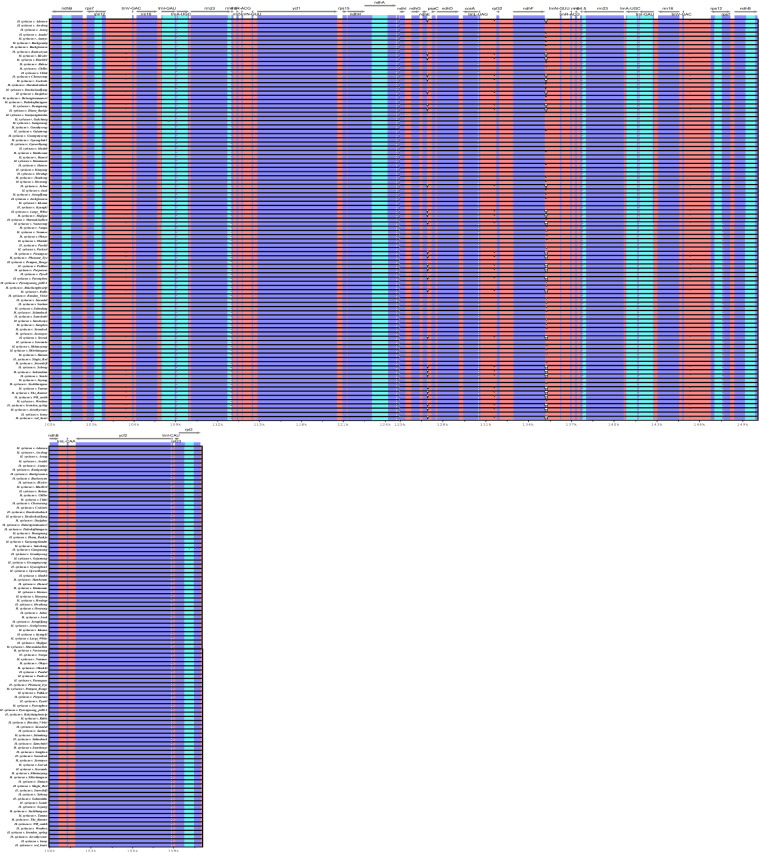
The 95 *H. syriacus* accessions mVISTA map, with the Gangneung chloroplast genome as the reference. The vertical scale represents the percentage of identity, ranging from 50% to 100%. The horizontal axis corresponds to the base sequence region. Red indicates non-coding sequences(CNS), blue indicates the exons of protein-coding genes and light green indicates untranslated regions(UTR) including tRNA or rRNA.

Structural similarity and gene distribution among the 95 chloroplast genomes were analyzed using mVISTA software in LAGAN mode with the default settings, with *H. syriacus* var. Baekdansim used as the reference^58–61^ (Fig. 3). This observation was consistent with the results from the pan-chloroplast genome analysis, where *H. syriacus* var. Russian Violet exhibits a significant deletion in specific regions.

**Fig. 4:**
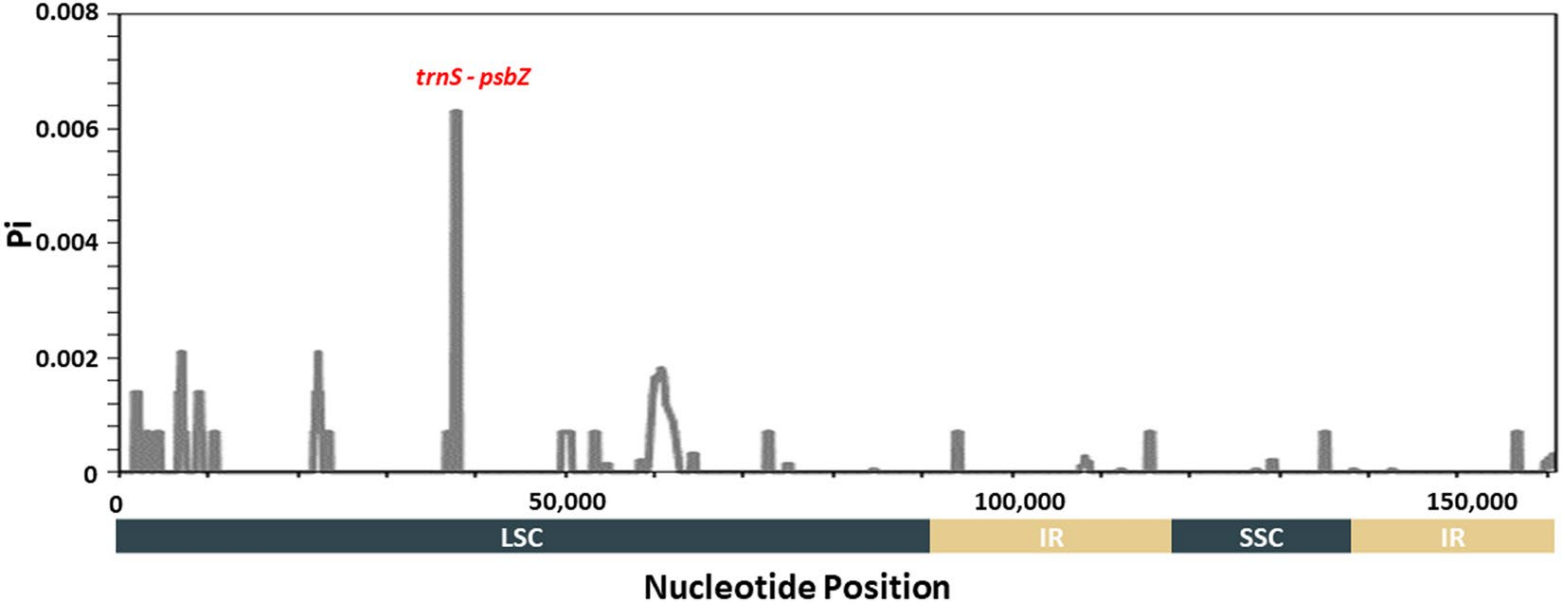
Nucleotide diversity in 95 *H. syriacus* chloroplast genomes. Sliding window analysis was performed with a window length of 600 bp and a step size of 100 bp. The x-axis represents nucleotide position, while the y-axis represents nucleotide diversity (Pi). Genes within the most hypervariable regions are highlighted in red.

Hypervariable regions within the chloroplast genome of *H. syriacus* were identified using DnaSP version 6 software^62^. A total of 95 *H. syriacus* chloroplast genomes were aligned using MAFFT^55^ with default parameters. Nucleotide diversity was calculated through sliding window analysis, with the window size set at 600 bp with a step size of 100 bp^22^ (Fig. 4). The inverted repeat regions tend to be more conservative than the single copy regions. The highest nucleotide diversity was identified in the *trnS*-*psbZ* region. This region has the potential for use as a DNA barcode to facilitate distinction among the *H. syriacus* cultivars.

## Data Records

A total of 94 raw reads obtained through Illumina sequencing have been deposited in the NCBI Sequence Read Archive under the accession number SRP464541^63^. The assembled chloroplast genome sequences, accompanied by their corresponding gene annotations for the 94 cultivars have been submitted to NCBI GenBank^64–157^ and are detailed in Table S1. Additionally, *H. syriacus* var. Gangneung has been deposited in the NCBI GenBank with the accession number OR619829^158^.

## Technical Validation

### Evaluation of chloroplast genome assembly

**Fig. 5:**
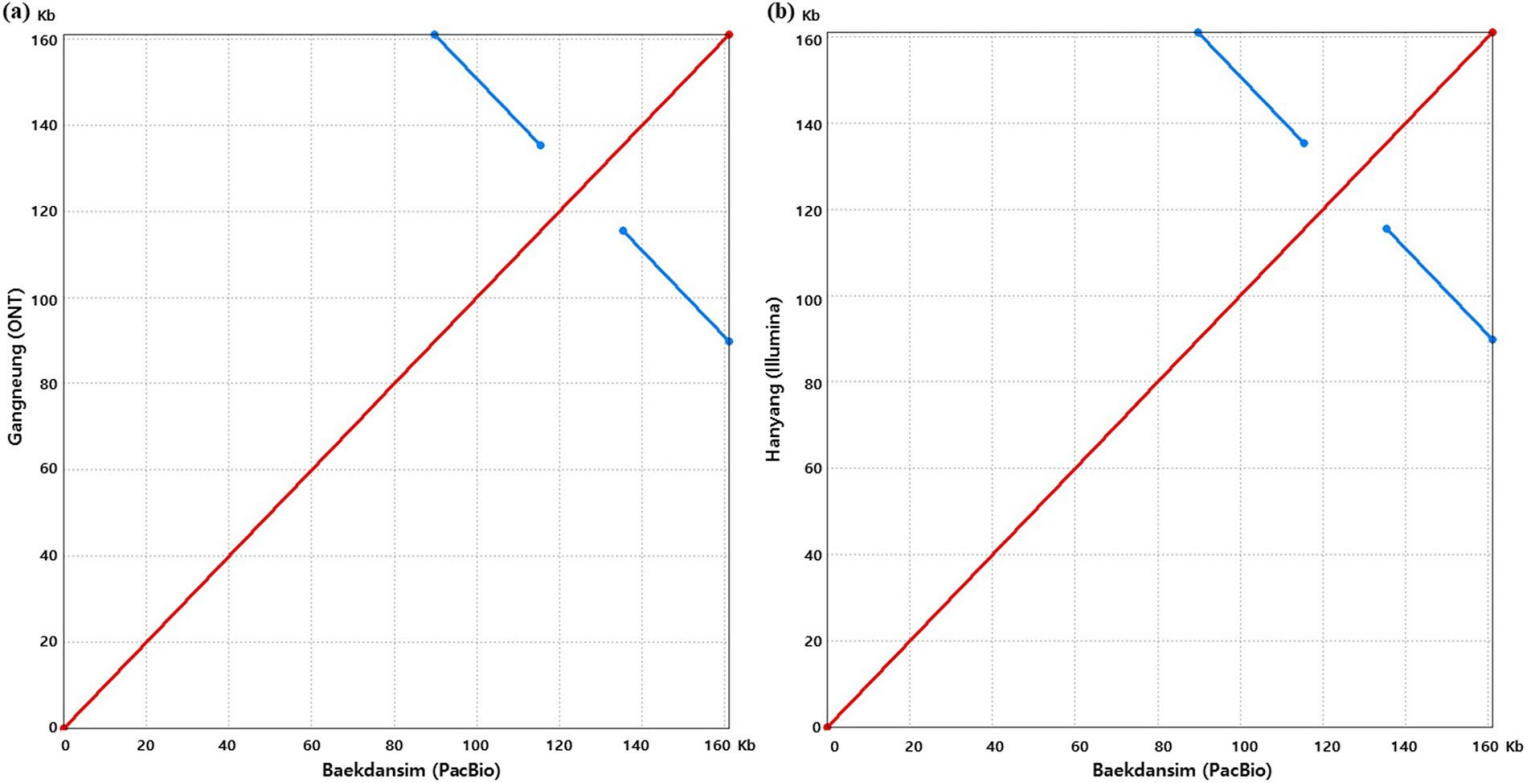
Pairwise comparative analysis of chloroplast genomes in various *H. syriacus* cultivars with *H. syriacus* var. Baekdansim using MUMmer plots. (**a**) Comparison of chloroplast genomes constructed using ONT and PacBio long-read sequencing platforms. (**b**) Comparison of chloroplast genomes constructed using Illumina short-read and PacBio long-read sequencing platforms. The red lines represent collinear sequences and the blue lines represent inverted sequences.

To evaluate the completeness of the chloroplast genome assembly, chloroplast reads were aligned to the chloroplast genome as described in the “Genome assembly and annotation” section. The lengths of the 95 assembled pseudo-molecules ranged from 160,231 bp to 161,041 bp, which is consistent with the observed chloroplast genome length in other members of the Malvaceae family^23–27^. Synteny analyses were conducted using MUMmer^159^ with the previously reported chloroplast genome of *H. syriacus* var. Baekdansim as the reference^30^. The dot plot revealed that the assembled genomes align cohesively with no major rearrangements observed (Fig. 5). Instead, the plot displayed inversions, represented by a blue line, corresponding to the chloroplast-specific inverted region.

### Evaluation of gene annotation

The accuracy of the gene annotations was meticulously evaluated by comparing them to the *H. syriacus* var. Baekdansim^61^ chloroplast genome. Any discrepancies identified were refined through manual curation. In total, 113 distinct genes were identified, including 79 protein-coding genes, 30 tRNA genes, and 4 rRNA genes (Table 2).

**Table 2:** Genes annotated in the chloroplast genome.

Gene category	Gene function	*Gene names*
Photosynthesis-related genes	Photosystem I	*psaA; psaB; psaC; psaI; psaJ*
	Photosystem II	*psbA; psbB; psbC; psbD; psbE; psbF; psbH; psbI; psbJ; psbK; psbL; psbM; psbT; psbZ*
	Cytochrome b/f complex	*petA; petB; petD; petG; petL; petN*
	ATP synthase	*atpA; atpB; atpE; atpF; atpH; atpI*
	NADH dehydrogenase	ndhA; ndhB*; ndhC; ndhD; ndhE; ndhF; ndhG; ndhH; ndhI; ndhJ; ndhK
	RubisCO large subunit	*rbcL*
	Photosystem assembly/stability Factors	*pafI; pafII; pbf1*
Self-replication-related genes	RNA polymerase	rpoA; rpoB; rpoC1*; rpoC2
	Ribosomal proteins (small subunit)	rps2; rps3; rps4; rps7*; rps8; rps11; rps12*; rps14; rps15; rps16; rps18; rps19
	Ribosomal proteins (large subunit)	rpl2*; rpl14; rpl16; rpl20; rpl22; rpl23*; rpl32; rpl33; rpl36
	tRNAs	trnA-UGC*; trnC-GCA; trnD-GUC; trnE-UUC; trnF-GAA; trnfM-CAU; trnG-GCC; trnG-UCC*; trnH-GUG; trnI-CAU*; trnI-GAU*; trnK-UUU; trnL-CAA*; trnL-UAA; trnL-UAG; trnM-CAU; trnN-GUU*; trnP-UGG; trnQ-UUG; trnR-ACG*; trnR-UCU; trnS-GCU; trnS-GGA; trnS-UGA; trnT-GGU; trnT-UGU; trnV-GAC*; trnV-UAC; trnW-CCA; trnY-GUA
	rRNAs	rrn5*; rrn4.5*; rrn16*; rrn23*
Other genes	Maturase	*matK*
	Envelope membrane protein	*cemA*
	Subunit of acetyl-CoA	*accD*
	C-type cytochrome synthesis gene	*ccsA*
	Protease	*clpP1*
	Translational initiation factor	*infA*
Unknown	Conserved hypothetical chloroplast reading frames	ycf1, ycf2*

*Indicates duplicated genes.

The gene repertoires were consistent across all 95 cultivars, with the only observed differences being related to specific gene loci details. Our results indicate that the gene repertoire was congruent with annotations commonly observed within the Malvaceae family^23,160,161^, with minor variations detected in the *pafI* (*ycf3*), *pafII* (*ycf4*), and *pbf1* (*psbN*) genes^162,163^.

### Supplementary information

Supplementary Information

Table S1

## Data Availability

All software used for data processing was implemented following the manual provided by the bioinformatic software cited in the method section. When specific parameters for the software were not detailed, the default settings were utilized.
