# Supplementary material for: Pan-chloroplast genomes for accession-specific marker development in Hibiscus syriacus
Source: Sci Data. 2024 Feb 27;11:246. doi: 10.1038/s41597-024-03077-7 (PMC10899175; doi:10.1038/s41597-024-03077-7)
Supplement: Supplementary file 1 — Supplementary Information [file 41597_2024_3077_MOESM1_ESM.docx]

**Supplementary Information**

**Pan-chloroplast genomes for accession-specific marker development in *Hibiscus syriacus***

Sangjin Go^1,2†^, Hyunjin Koo^1†^, Minah Jung^1^, Seongmin Hong^3^, Gibum Yi^3^ & Yong-Min Kim^1,2,4*^

^1^Plant Systems Engineering Research Center, Korea Research Institute of Bioscience and Biotechnology (KRIBB), Daejeon 34141, Republic of Korea

^2^Department of Bioinformatics, KRIBB School of Bioscience, Korea University of Science and Technology (UST), Daejeon 34141, Republic of Korea

^3^Department of Bio-Environmental Chemistry, College of Agriculture and Life Sciences, Chungnam National University, Daejeon 34134, Republic of Korea

^4^Digital Biotech Innovation Center, Korea Research Institute of Bioscience and Biotechnology (KRIBB), Daejeon 34141, Republic of Korea

^†^These authors contributed equally to this work.

^*^Correspondence should be addressed to Y.-M. K. (ymkim@kribb.re.kr)

**Table of Contents**

Table S1 ∙∙∙∙∙∙∙∙∙∙∙∙∙∙∙∙∙∙∙∙∙∙∙∙∙∙∙∙∙∙∙∙∙∙∙∙∙∙∙∙∙∙∙∙∙∙∙∙∙∙∙∙∙∙∙∙∙∙∙∙∙∙∙∙∙∙∙∙∙∙∙∙∙∙∙∙∙∙∙∙∙∙∙∙∙∙∙∙∙∙∙∙∙∙∙∙∙∙∙∙∙∙∙ 2

**Table S1. Sequence Read Archive and GenBank accession numbers of 94 *H. syriacus* cultivars.**

| Sample name | Cultivar | Read count | Total bases | Sequence Read Archive accession number | GenBank  accession  number |
| --- | --- | --- | --- | --- | --- |
| HS_ch_01 | *Adenseu* | 10,924,766 | 1,617,040,338 | SRX21992312 | OR350231.1 |
| HS_ch_02 | *An-dong* | 14,093,288 | 2,083,322,344 | SRX21992313 | OR164399.1 |
| HS_ch_03 | *Arang* | 14,257,218 | 2,119,434,764 | SRX21992324 | OR397843.1 |
| HS_ch_04 | *Asadal* | 13,490,094 | 1,997,749,767 | SRX21992335 | OR397844.1 |
| HS_ch_05 | *Asanyo* | 17,159,718 | 2,546,357,978 | SRX21992346 | OR397845.1 |
| HS_ch_06 | *Bicolor* | 15,531,672 | 2,291,323,839 | SRX21992357 | OR397846.1 |
| HS_ch_07 | *Bluebird* | 10,375,324 | 1,524,640,066 | SRX21992368 | OR397847.1 |
| HS_ch_08 | *Bulsae* | 8,111,146 | 1,200,868,300 | SRX21992379 | OR397848.1 |
| HS_ch_09 | *Chilbo* | 17,783,984 | 2,637,878,938 | SRX21992390 | OR397849.1 |
| HS_ch_10 | *Chini* | 19,953,880 | 2,942,187,947 | SRX21992401 | OR397850.1 |
| HS_ch_11 | *Chossarang* | 17,263,562 | 2,555,357,403 | SRX21992314 | OR397851.1 |
| HS_ch_12 | *Coelestis* | 15,583,648 | 2,308,440,991 | SRX21992315 | OR397852.1 |
| HS_ch_13 | *Daisengionmamori* | 11,160,456 | 1,646,341,625 | SRX21992316 | OR397853.1 |
| HS_ch_14 | *Daitokujihanagasa* | 12,506,214 | 1,848,607,791 | SRX21992317 | OR397854.1 |
| HS_ch_15 | *Gaeryangdansim* | 11,488,542 | 1,701,382,223 | SRX21992318 | OR397855.1 |
| HS_ch_16 | *Geunhyeong* | 14,059,518 | 2,090,602,281 | SRX21992319 | OR397856.1 |
| HS_ch_17 | *Gojumong* | 9,812,902 | 1,452,614,766 | SRX21992320 | OR397857.1 |
| HS_ch_18 | *Gwangmyeong* | 11,374,564 | 1,683,838,120 | SRX21992321 | OR397858.1 |
| HS_ch_19 | *Gyeongbuk1* | 9,723,938 | 1,434,968,092 | SRX21992322 | OR397859.1 |
| HS_ch_20 | *Gyewolhyang* | 11,296,944 | 1,659,391,093 | SRX21992323 | OR397860.1 |
| HS_ch_21 | *Hanbit* | 21,895,204 | 3,240,089,497 | SRX21992325 | OR397861.1 |
| HS_ch_22 | *Hanboram* | 7,482,664 | 1,107,718,723 | SRX21992326 | OR397862.1 |
| HS_ch_23 | *Haneol* | 12,875,510 | 1,906,815,031 | SRX21992327 | OR397863.1 |
| HS_ch_24 | *Hanmaum* | 21,928,404 | 3,247,253,661 | SRX21992328 | OR397864.1 |
| HS_ch_25 | *Hanseo* | 13,865,994 | 2,050,950,968 | SRX21992329 | OR397865.1 |
| HS_ch_26 | *Hanyang* | 14,510,676 | 2,148,061,315 | SRX21992330 | OR397866.1 |
| HS_ch_27 | *Hwahap* | 16,468,512 | 2,418,432,282 | SRX21992331 | OR397867.1 |
| HS_ch_28 | *Hwahong* | 11,880,928 | 1,746,397,642 | SRX21992332 | OR397868.1 |
| HS_ch_29 | *Jaok* | 9,441,978 | 1,401,093,604 | SRX21992333 | OR397869.1 |
| HS_ch_30 | *Kkoma* | 22,803,884 | 3,374,129,455 | SRX21992334 | OR397870.1 |
| HS_ch_31 | *Kyungki* | 10,742,604 | 1,593,548,539 | SRX21992336 | OR397871.1 |
| HS_ch_32 | *Large_White* | 19,860,224 | 2,934,453,152 | SRX21992337 | OR397872.1 |
| HS_ch_33 | *Mujigae* | 11,428,002 | 1,696,897,469 | SRX21992338 | OR397873.1 |
| HS_ch_34 | *Murasakisaiben* | 12,400,294 | 1,832,647,251 | SRX21992339 | OR397874.1 |
| HS_ch_35 | *Naesarang* | 12,904,688 | 1,906,929,680 | SRX21992340 | OR397875.1 |
| HS_ch_36 | *Nanpa* | 10,037,582 | 1,489,455,688 | SRX21992341 | OR397876.1 |
| HS_ch_37 | *Nunmoe* | 14,540,116 | 2,157,005,979 | SRX21992342 | OR397877.1 |
| HS_ch_38 | *Oknyo* | 16,554,750 | 2,449,326,235 | SRX21992343 | OR397878.1 |
| HS_ch_39 | *Oktokki* | 17,303,190 | 2,560,981,092 | SRX21992344 | OR397879.1 |
| HS_ch_40 | *Paedal* | 24,503,620 | 3,619,049,655 | SRX21992345 | OR397880.1 |
| HS_ch_41 | *Paeksol* | 13,860,442 | 2,048,202,957 | SRX21992347 | OR397881.1 |
| HS_ch_42 | *Parangsae* | 9,201,658 | 1,359,945,733 | SRX21992348 | OR397882.1 |
| HS_ch_43 | *Pheasant_Eye* | 21,570,402 | 3,202,415,566 | SRX21992349 | OR397883.1 |
| HS_ch_44 | *Pompon_Rouge* | 14,262,034 | 2,099,680,030 | SRX21992350 | OR397884.1 |
| HS_ch_45 | *Pulkkot* | 12,611,072 | 1,866,433,580 | SRX21992351 | OR397885.1 |
| HS_ch_46 | *Purpureus* | 12,303,722 | 1,814,248,345 | SRX21992352 | OR397886.1 |
| HS_ch_47 | *Pyeli* | 10,233,800 | 1,517,551,387 | SRX21992353 | OR397887.1 |
| HS_ch_48 | *Pyeonghwa* | 19,163,156 | 2,838,271,812 | SRX21992354 | OR397888.1 |
| HS_ch_49 | *Rubis* | 8,163,778 | 1,203,117,114 | SRX21992355 | OR397889.1 |
| HS_ch_50 | *Russian_Violet* | 7,704,918 | 1,136,673,388 | SRX21992356 | OR397890.1 |
| HS_ch_51 | *Saeasadal* | 14,897,962 | 2,204,063,854 | SRX21992358 | OR397891.1 |
| HS_ch_52 | *Saehan* | 20,191,516 | 2,989,338,564 | SRX21992359 | OR397892.1 |
| HS_ch_53 | *Salmabaek* | 17,162,908 | 2,545,798,940 | SRX21992360 | OR397893.1 |
| HS_ch_54 | *Samchulri* | 14,762,538 | 2,179,944,143 | SRX21992361 | OR397894.1 |
| HS_ch_55 | *Sanchonye* | 12,064,768 | 1,782,986,638 | SRX21992362 | OR397895.1 |
| HS_ch_56 | *Seondeok* | 11,132,564 | 1,656,133,011 | SRX21992363 | OR397896.1 |
| HS_ch_57 | *Seonnyeo* | 11,900,092 | 1,756,248,697 | SRX21992364 | OR397897.1 |
| HS_ch_58 | *Seorak* | 6,982,520 | 1,034,309,551 | SRX21992365 | OR397898.1 |
| HS_ch_59 | *Serenade* | 20,121,218 | 2,967,775,712 | SRX21992366 | OR397899.1 |
| HS_ch_60 | *Shintaeyang* | 14,485,060 | 2,138,669,491 | SRX21992367 | OR397900.1 |
| HS_ch_61 | *Shirohanagasa* | 17,831,082 | 2,644,228,735 | SRX21992369 | OR397901.1 |
| HS_ch_62 | *Single_Red* | 12,718,566 | 1,876,739,254 | SRX21992370 | OR397902.1 |
| HS_ch_63 | *Snowdrift* | 9,249,076 | 1,363,170,157 | SRX21992371 | OR397903.1 |
| HS_ch_64 | *Sobong* | 22,697,624 | 3,351,958,409 | SRX21992372 | OR397904.1 |
| HS_ch_65 | *Soltanshim* | 18,177,638 | 2,682,280,000 | SRX21992373 | OR397905.1 |
| HS_ch_66 | *Sonde* | 7,779,904 | 1,150,036,730 | SRX21992374 | OR397906.1 |
| HS_ch_67 | *Soyang* | 14,290,562 | 2,111,300,934 | SRX21992375 | OR397907.1 |
| HS_ch_68 | *Suchihanagasa* | 10,730,900 | 1,587,824,064 | SRX21992376 | OR397908.1 |
| HS_ch_69 | *Tamna* | 6,450,616 | 955,440,822 | SRX21992377 | OR397909.1 |
| HS_ch_70 | *The_Banner* | 13,066,380 | 1,933,212,792 | SRX21992378 | OR397910.1 |
| HS_ch_71 | *Wonhwa* | 7,181,886 | 1,062,312,167 | SRX21992380 | OR397911.1 |
| HS_ch_72 | *Baekgeunip* | 9,416,528 | 1,382,467,535 | SRX21992381 | OR625140.1 |
| HS_ch_73 | *Baekgiwonsu* | 6,582,630 | 964,367,590 | SRX21992382 | OR625141.1 |
| HS_ch_74 | *Baeksoryun* | 7,211,818 | 1,058,430,839 | SRX21992383 | OR625142.1 |
| HS_ch_75 | *bredon_spring* | 8,675,430 | 1,273,545,864 | SRX21992384 | OR625143.1 |
| HS_ch_76 | *Daedeoksaback* | 5,771,614 | 847,541,126 | SRX21992385 | OR625144.1 |
| HS_ch_77 | *Daedeoksailjung* | 7,286,690 | 1,070,134,579 | SRX21992386 | OR625145.1 |
| HS_ch_78 | *Diana_Baekjo* | 8,717,086 | 1,281,972,832 | SRX21992387 | OR625148.1 |
| HS_ch_79 | *dorothycranc* | 8,403,658 | 1,232,307,340 | SRX21992388 | OR625149.1 |
| HS_ch_80 | *Gakchang* | 6,114,078 | 897,872,424 | SRX21992389 | OR625150.1 |
| HS_ch_81 | *Jeogiljung* | 10,806,636 | 1,585,350,437 | SRX21992391 | OR625153.1 |
| HS_ch_82 | *Jeokgiwonsu* | 5,956,514 | 873,484,456 | SRX21992392 | OR625154.1 |
| HS_ch_83 | *lenny* | 7,231,840 | 1,061,272,231 | SRX21992393 | OR625155.1 |
| HS_ch_84 | *Rakchanghwarip* | 12,179,410 | 1,788,247,811 | SRX21992394 | OR625157.1 |
| HS_ch_85 | *Sangbon* | 8,138,074 | 1,189,352,020 | SRX21992395 | OR625160.1 |
| HS_ch_86 | *WR_smith* | 7,811,808 | 1,147,186,630 | SRX21992396 | OR625161.1 |
| HS_ch_87 | *Hwarang* | 3,800,466 | 555,529,682 | SRX21992397 | OR625151.1 |
| HS_ch_88 | *Saimdang* | 7,919,568 | 1,162,917,091 | SRX21992398 | OR625159.1 |
| HS_ch_89 | *Daejabae* | 7,056,906 | 1,029,121,245 | SRX21992399 | OR625146.1 |
| HS_ch_90 | *Deungnang* | 7,233,996 | 1,062,643,538 | SRX21992400 | OR625147.1 |
| HS_ch_91 | *Jabae* | 9,453,164 | 1,379,816,932 | SRX21992402 | OR625152.1 |
| HS_ch_92 | *Pyeongseong_ps80-1* | 11,408,356 | 1,675,286,558 | SRX21992403 | OR625156.1 |
| HS_ch_93 | *red_heart* | 8,297,002 | 1,218,846,381 | SRX21992404 | OR625158.1 |
| HS_ch_94 | *Simsan* | 9,453,726 | 1,398,710,934 | SRX21992405 | OR619828.1 |
